# M448R and MGF505-7R: Two African Swine Fever Virus Antigens Commonly Recognized by ASFV-Specific T-Cells and with Protective Potential

**DOI:** 10.3390/vaccines9050508

**Published:** 2021-05-14

**Authors:** Laia Bosch-Camós, Elisabet López, Javier Collado, María J. Navas, Miguel Blanco-Fuertes, Sonia Pina-Pedrero, Francesc Accensi, Maria Luisa Salas, Egbert Mundt, Veljko Nikolin, Fernando Rodríguez

**Affiliations:** 1 IRTA, Centre de Recerca en Sanitat Animal (CReSA, IRTA), Campus de la Universitat Autònoma de Barcelona, 08193 Bellaterra, Spain; laia.bosch@irta.cat (L.B.-C.); elisabeth.lopezf@gmail.com (E.L.); mariajesus.navas@irta.cat (M.J.N.); miguel.blanco@irta.cat (M.B.-F.); sonia.pina@irta.cat (S.P.-P.); francesc.accensi@irta.cat (F.A.); 2 OIE Collaborating Centre for the Research and Control of Emerging and Re-Emerging Swine Diseases in Europe (IRTA-CReSA), 08193 Bellaterra, Spain; 3 Departament de Biologia Cellular, Fisiologia i Immunologia, Campus de la Universitat Autònoma de Barcelona, 08193 Bellaterra, Spain; javiercolladomiguens@gmail.com; 4 UAB, Centre de Recerca en Sanitat Animal (CReSA, IRTA-UAB), Campus de la Universitat Autònoma de Barcelona, 08193 Bellaterra, Spain; 5 Centro de Biología Molecular Severo Ochoa, Consejo Superior de Investigaciones Científicas and Universidad Autònoma de Madrid, 28049 Madrid, Spain; mlsalas@cbm.csic.es; 6 Boehringer Ingelheim Veterinary Research Center (BIVRC) GmbH & Co. KG, 30559 Hannover, Germany; egbert.mundt@boehringer-ingelheim.com (E.M.); veljko.nikolin@boehringer-ingelheim.com (V.N.)

**Keywords:** African swine fever, antigen discovery, T-cells, DNA immunization, live attenuated virus, protection, immunopeptidomics

## Abstract

African swine fever (ASF) is today′s number one threat for the global swine industry. Neither commercial vaccine nor treatment is available against ASF and, thus far, only live attenuated viruses (LAV) have provided robust protection against lethal ASF virus (ASFV) challenge infections. Identification of ASFV proteins inducing protective immune responses is one of the major challenges to develop safer and efficient subunit vaccines. Immunopeptidomic studies recently performed in our laboratory allowed identifying ASFV antigens recognized by ASFV-specific CD8^+^ T-cells. Here, we used data from the SLAI-peptide repertoire presented by a single set of ASFV-infected porcine alveolar macrophages to generate a complex DNA vaccine composed by 15 plasmids encoding the individual peptide-bearing ORFs. DNA vaccine priming improved the protection afforded by a suboptimal dose of the BA71ΔCD2 LAV given as booster vaccination, against Georgia2007/1 lethal challenge. Interestingly, M448R was the only protein promiscuously recognized by the induced ASFV-specific T-cells. Furthermore, priming pigs with DNA plasmids encoding M488R and MGF505-7R, a CD8^+^ T-cell antigen previously described, confirmed these two proteins as T-cell antigens with protective potential. These studies might be useful to pave the road for designing safe and more efficient vaccine formulations in the future.

## 1. Introduction

African swine fever (ASF), a pig hemorrhagic disease of obligatory notification to the World Organization for Animal Health (OIE), is currently one of the major threats to domestic pigs worldwide. While ASF has been enzootic in many Sub-Saharan countries since its discovery in 1921, the last occurrence of ASF virus (ASFV) in Georgia in 2007 has provoked its global expansion, starting first in the Caucasian region and Russia and reaching countries of the European Union in 2014 [1,2]. ASFV in this area has circulated between domestic pigs and wild boars, equally susceptible to ASFV, complicating the control of the disease [3]. The expansion of ASF in Asia and Oceania has been much faster than in Europe since it was first detected in China in Summer 2018 [4,5], mostly mediated by human trading activities, with wild boars playing a marginal role thus far. Due to the lack of commercial vaccines and therapeutics, classic sanitary methods remain as the only control strategy for ASF. These include early diagnosis followed by stamping out of the infected and potentially in contact animals, extensive disinfection and rigorous biosecurity protocols on farms [6]. Confirmation of ASF represents major trade restrictions and serious economic losses for the affected regions [7,8]. Therefore, developing safe and efficient vaccines against ASF is imperative if aiming to control and eradicate the disease.

The complexity of ASFV, a large, multi-enveloped and multi-capsid, icosahedral virus with a dsDNA genome of about 180 kbp in length [9], has complicated the generation of safe and efficient vaccines. As described in the latest blueprint published by the European Commission, live attenuated viruses (LAVs) are the most plausible ASF-vaccine option in the short term [10]. The only data indicating protection from challenge infection available today against the pandemic ASFV strain, currently circulating in Asia and Europe, come from the use of naturally attenuated virus strains [11,12] or recombinant LAVs [13,14,15,16,17,18]. Despite their protective abilities, LAVs have to improve their safety and DIVA capabilities to be commercially viable in ASF-free areas. While recognizing the key role that future licensed ASF LAVs will play to control ASF in endemic and infected areas and/or in emergencies, it is indispensable to continue our research on safer ASF vaccine alternatives for global use.

The presence of more than 150 open reading frames (ORFs) in the ASFV genome [9,19,20], together with the lack of protection correlates, hampers the design of rational subunit vaccine formulations. As a good example, and even though the crucial role of CD8^+^ T-cells in protection against ASFV has been established [21], the relevant antigens specifically stimulating this cell subset and inducing protective responses have not been fully determined yet. Thus, subunit vaccine formulations used in experimental setups have only conferred partial protection against ASF, and, unfortunately, these results have not been reproduced against the Georgia2007/1 pandemic virus currently circulating [22]. Gaining knowledge on this critical gap is essential to allow the rational design of future subunit vaccines. Due to the failure of previous formulations using DNA vaccination alone against Georgia2007/1 and aiming to increase the chances to discover new T-cell determinants with protective potential against Georgia2007/1, we decided to perform two independent in vivo experiments following a heterologous prime–boost regime. Therefore, here we first showed that DNA immunization of pigs with a cocktail of 15 plasmids encoding one ASFV protein eac, enhanced the protection afforded by a suboptimal dose of BA71∆CD2 [14] and confirmed M448R as the main target for the T-cells induced. The proteins were selected based on SLAI binding peptides identified using porcine alveolar macrophages (PAMs) infected with ASFV in vitro. A second immunization experiment was performed by priming pigs with a mix of pCMV-Ub-M448R and pCMV-Ub-MGF505-7R only, encoding M488R and MGF505-7R, previously identified as commonly recognized by CD8^+^ T-cells [23], to identify their protective potential. We finally discuss the possibilities of these methodologies for antigen discovery and designing future subunit vaccines.

## 2. Materials and Methods 

### 2.1. Cells

Porcine primary cells were collected from ear tissue samples and fibroblast monolayers were obtained following previously described protocols [24]. Porcine peripheral blood mononuclear cells (PBMCs) were isolated from whole blood using Histopaque-1077 density gradient solution (Sigma-Aldrich, Saint Louis, MO, USA) and maintained in RPMI 1640 medium (Gibco, Thermo Fisher Scientific, Waltham, MA, USA) supplemented with 10% heat-inactivated FBS (GE HealthCare, Chicago, IL, USA), 100 IU/mL of penicillin (Invitrogen, Carlsbad, CA, USA), 100 μg/mL of streptomycin (Invitrogen) and 2 mM L-glutamine (Invitrogen). Fifty micromolar β-mercaptoethanol (Sigma-Aldrich, Saint Louis, MO, USA) was added to the medium to help maintain reducing conditions during the ELISpot assays. Rabbit kidney epithelial RK13 cells were cultured in DMEM supplemented with 10% FBS, 100 IU/mL of penicillin, 100 μg/mL of streptomycin and 2 mM L-glutamine. 

### 2.2. African Swine Fever Virus Strains

BA71ΔCD2 is a LAV lacking the CD2v gene (EP402R), obtained by homologous recombination from the parental virulent BA71 ASFV strain [14]. Immunization with BA71ΔCD2 protects pigs against homologous and heterologous ASFV lethal challenge in a dose dependent manner. Georgia2007/1 is the pandemic virulent strain of ASFV currently circulating in Asia and Europe and was kindly provided by Dr. Linda Dixon (The Pirbright Research Institute, UK).

### 2.3. Identification of SLAI-Bound Peptides 

First, 5 × 10^6^ PAMs/well were seeded in a 6-well plate for ASFV infection, using a multiplicity of infection (MOI) of 0.1. Cells were harvested by scrapping when cytopathic effect was evident. Cells were sedimented by centrifugation and obtained cell pellets were frozen at −80 °C until further use for affinity purification of SLAI molecules followed by liquid chromatography coupled to mass spectrometry (LC-MS/MS) as previously described [23]. 

### 2.4. Plasmids Encoding ASFV Genes

Fifteen Georgia2007/1 ORFs encoding B475L, B602L, CP2475L (partial), D339L, DP238L, EP424R, H339R, I226R, I243L, I73R, I9R, K145R, M448R, MGF505-1R and MGF505-3R (GenBank accession number FR682468) were individually cloned into the pCMV plasmid (Clontech Laboratories, Inc., Mountain View, CA, USA). The plasmid pCMV-Ub-MGF505-7R was previously used to define MGF505-7R as an ASFV antigen recognized by ASFV-specific CD8^+^ T-cells [23]. Each ORF was cloned in frame with ubiquitin at the N-terminus with the aim to improve proteasome degradation and subsequent CD8^+^ T-cell induction [25]. In addition, a FLAG-tag sequence was located at the C-terminal end to facilitate the detection of protein expression [26]. Protein expression was analyzed by anti-FLAG-tag immunofluorescence in transfected RK13 cells. Transfection of RK13 cells was performed using Lipofectamine 3000 transfection kit (Invitrogen) according to the manufacturers’ instructions. After paraformaldehyde fixation and subsequent Tween-20 permeabilization, cell monolayers were incubated with AlexaFluor 488-conjugated anti-FLAG-tag monoclonal antibody (MA1-142-A488, Invitrogen) diluted 1:100. Hoechst 33342 (Thermo Fisher Scientific, Waltham, MA, USA) was used for nucleus staining. Cells were finally examined by fluorescence microscopy. 

### 2.5. In Vivo Experimental Design

**Figure 1:**
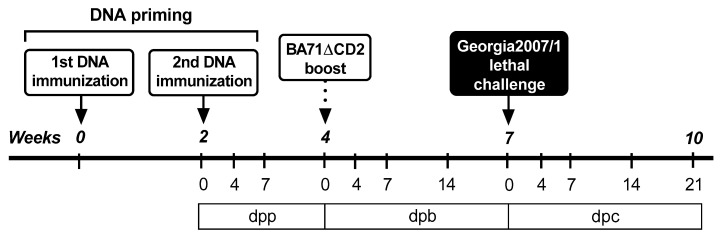
Schematic representation of the in vivo experimental designs. The two in vivo experiments performed in this study followed an identical scheme but priming with different plasmid combinations: either 15 clones in Experiment 1 or the combination of two plasmids (pCMV-Ub-M448R + pCMV-Ub-MGF505-7R) in Experiment 2. Groups of five pigs were primed twice two weeks apart using the indicated DNA plasmid mixes and boosted with 10^3^ PFU of BA71∆CD2. Three weeks after the boost, pigs were challenged with a lethal dose of Georgia2007/1. Samples were taken at different days post priming (dpp), post boost (dpb) or post challenge (dpc).

Two independent experiments were performed using groups of five four-week-old male piglets (Landrace × Large White), housed in experimental boxes of the biosafety level 3 facilities at IRTA-CReSA (Barcelona, Spain). After 7 days of acclimatization, all pigs were immunized using a heterologous prime–boost regime, priming twice with plasmid DNA encoding ASFV proteins (DNA priming) and subsequently boosted with 10^3^ plaque-forming units (PFU) of BA71ΔCD2, a recombinant live attenuated vaccine dose previously described as partially protective [14]. For DNA priming, pigs were immunized with either the empty pCMV-Ub plasmid (negative control) or a mixture of the corresponding recombinant plasmids. Pigs were immunized twice with 0.6 mg of total endotoxin-free DNA (Qiagen, Hilden, Germany) in 1.5 mL saline, two weeks apart, following the previously described protocol [27]. Two weeks after the second DNA immunization, all pigs were intramuscularly inoculated (heterologous boost) with 10^3^ PFU of BA71ΔCD2. Finally, pigs were challenged intramuscularly with a lethal dose of 10^3^ gene equivalent copies (GEC) of Georgia2007/1, three weeks after last immunization (Figure 1). Blood samples and nasal swabs were taken at different time points after: DNA primary vaccination (0, 4 and 7 days post primary vaccination (dpp)), BA71ΔCD2 boost (0, 4, 7 and 14 days post booster vaccination (dpb)) and Georgia2007/1 challenge (0, 4, 7, 14 and 21 days postchallenge infection (dpc)). Rectal temperatures were monitored daily, as well as ASF typical clinical signs, including behavior, body condition (prominence of vertebrae and ribs), cyanosis, digestive and respiratory signs. Each parameter was scored from 0 to 3 according to the severity (0: normal; 1: mild; 2: moderate; 3: severe). Post-mortem examinations were carried out to confirm or discard the presence of ASF typical pathological lesions [28]. Animal care and procedures were carried out in accordance with the guidelines of the Good Experimental Practices and under the supervision of the Commission of Animal Experimentation of Generalitat de Catalunya (approval code CEA-OH/9212/2). As mentioned above, two independent in vivo experiments were performed priming pigs with different plasmid compositions. In the first experiment, pigs were primed with the cocktail of 15 plasmids, and, in the second experiment, pigs were primed with a mix of pCMV-Ub-M448R and pCMV-Ub-MGF505-7R, following the procedure described above. 

### 2.6. Analytical Assays

DNA was obtained from sera and nasal swab-PBS suspensions (NucleoSpin Blood kit; Macherey-Nagel, Düren, German) and ASFV-specific DNA was quantified by real-time PCR (qPCR), using methods previously described in our laboratory [29]. Results were expressed as log_10_ numbers of GEC per mL of sera or nasal swab-PBS suspension. The limit of detection of the assay was established at 10^3^ GEC/mL. For comparative purposes, our qPCR technique has previously shown more reproducible results in sera than in whole blood. Therefore, virus in sera and no viremia was measured, being aware that the amount of virus present in blood would be around 1 log higher in magnitude. 

ASFV-specific immunoglobulin G (IgG) in pig sera were detected by the OIE-approved indirect ELISA assay based on the use of soluble extracts from ASFV-infected cells [30]. Plates were read at a wavelength of 450 nm and results from individual pigs were expressed as optical density (OD) values.

ASFV specific T-cell responses were assessed for each individual pig at the indicated times after immunization and after Georgia2007/1 challenge by IFNΥ ELISpot assay using PBMCs from immunized pigs as previously reported [29]. As specific stimuli, PBMCs were incubated overnight with 10^5^ PFU BA71ΔCD2 per well (multiplicity of infection of 0.2). RPMI and 10 µg/mL phytohemagglutinin-M (PHA-M, Sigma-Aldrich, Saint Louis, MO, USA) were used as negative and positive controls, respectively. The frequency of specific IFNΥ-secreting cells (IFNΥ-SC) represented in the graphs is the mean of two replicates, subtracting the counts in the negative control wells. Three hundred spots/well was considered the limit of our assay resolution (wells with more than 300 spots received a score of 300).

When fibroblastoid cells were used as antigen presenting cells (APCs) in the ELISpot assay, the ratio used was one APC to five autologous PBMCs, and plasmid transfection was performed by electroporation using the Neon Transfection System 10 µL Kit (Invitrogen, Carlsbad, CA, USA), as previously described [23]. The number of spots in a control well using fibroblastoid cells transfected with the empty pCMV-Ub plasmid, which never exceeded 10, was subtracted from the specific IFNΥ-SC represented in the graphs. 

### 2.7. Statistical Analysis

For transparency, data from individual animals (always groups of five pigs each) are provided. Complementarily, statistical analysis was inferred between groups applying a standard lineal model (SliM) at different time points while there were enough pigs alive. Thus, no statistical analysis could be performed from the time in which control groups counted with only one surviving pig (in both experiments). This analysis was done using RStudio software [31] with tidyverse and survival packages [32,33]. Statistical significance was set at *p* < 0.05. Sample size is one of the main factors affecting the power of statistical tests, therefore due to lack of enough surviving pigs in the control group (only one out of five in each experiment) analysis were limited. 

## 3. Results

### 3.1. DNA Immunization with a Cocktail of Plasmids Encoding 15 ASFV Pre-Selected Proteins Improves Protection against Georgia2007/1 Challenge Infection

To confirm the potential of our immunopeptidomic assays to identify potentially protective ASFV antigens, we infected a randomly selected PAM sample from our cell culture collection with BA71∆CD2, at a MOI of 0.1. Fifty-four hours after infection, cells were lysed, SLAI-peptide complexes were immunoprecipitated, the SLAI-bound peptides were eluted and subjected to LC-MS/MS as recently described [23]. As shown in Table 1, 17 individual ASFV peptides were identified, all of them between 8 and 12 amino acids in length and belonging to 15 different antigens (Table 1). The corresponding ORFs were cloned into the pCMV plasmid, under the control of an immediate early promoter of human cytomegalovirus for eukaryotic expression. Each ORF was cloned in frame with ubiquitin to optimize their SLAI antigen presentation and the induction of specific CD8+ T-cell responses [29,34] and with the FLAG-tag peptide in their C-terminal end to confirm their expression. Thus, before any in vivo experiment, the expression of each protein was tested by transient transfection of RK13 cells and anti-FLAG tag immunofluorescence (Table 1). 

**Figure 2:**
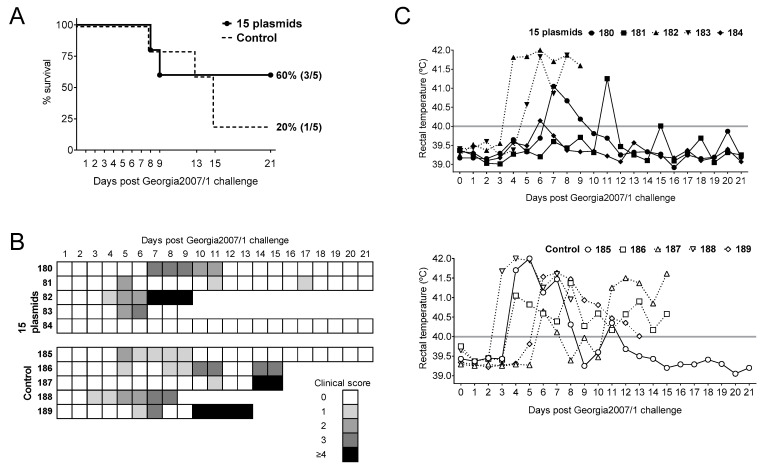
Priming pigs with 15 plasmids improves the protection induced by suboptimal BA71ΔCD2 immunization against lethal Georgia2007/1 challenge. Pigs were immunized twice with either the empty pCMV-Ub plasmid (Control) or the 15 plasmids and next boosted with 10^3^ PFU BA71ΔCD2. Two weeks later, all pigs were challenged with a lethal dose of Georgia2007/1 and (**A**) deaths, (**B**) ASF-compatible clinical signs and (**C**) rectal temperature from 15 plasmids (top) and control (bottom) groups were recorded daily. Solid lines represent animals that survived the challenge while dashed lines symbolize animals that succumbed the challenge.

Once the expression of the appropriate protein was confirmed, a group of five pigs was immunized twice two weeks apart with the 15 plasmids cocktail (priming). Five pigs receiving pCMV-Ub served as control group. Two weeks later, all pigs were boosted with 10^3^ PFU of BA71ΔCD2, and three weeks later were challenged with a lethal dose of Georgia2007/1 (Figure 1). As expected from previous results using 10^3^ PFU of BA71ΔCD2 [14], only one out of the five control pigs (20%) survived the lethal challenge with Georgia2007/1 (Figure 2A). Conversely, three out of five (60%) pigs primed with the 15 recombinant plasmids and boosted with 10^3^ PFU BA71ΔCD2 survived the lethal challenge with Georgia2007/1 (Figure 2A).

All pigs that succumbed to Georgia2007/1 challenge suffered typical ASF signs, evident from Days 4–5 post-challenge (pc) (Figure 2B), independently of the group. These included lethargy, general body condition, digestive signs, respiratory signs, cyanosis and fever (Figure 2C). Interestingly, the only control pig surviving Georgia2007/1 challenge, Pig 185, showed high fever during Days 4–9 pc, while immunized pigs only showed brief and milder peaks of fever, with Pig 184 showing no apparent clinical signs typical for ASF. 

**Figure 3:**
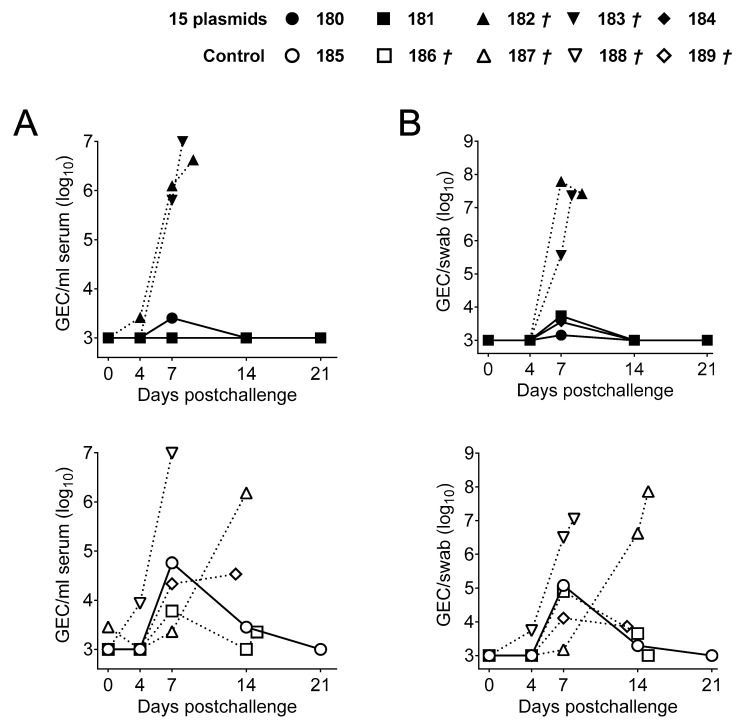
ASFV virus titers in sera and nasal swabs found in pigs after Georgia2007/1 challenge. (**A**) ASFV titers as indicated by GEC/mL found in sera and (**B**) nasal swabs analyzed by qPCR at different time points post challenge in the 15 plasmids group (top) and the control group (bottom). Solid lines represent animals that survived the challenge while dashed lines indicate animals that succumbed to the challenge.

Clinical signs and rectal temperature matched almost perfectly the viral loads found both in sera (Figure 3A) and nasal swabs (Figure 3B). As expected, pigs succumbing Georgia2007/1 challenge showed detectable ASFV as early as at Day 4 pc. All surviving pigs showed a strong reduction of virus in both samples as indicated by decreased GEC. Thus, the only surviving pig from the control group (Pig 185) showed a maximum of 10^5^ GEC of ASFV detectable between Days 7 and 14 pc, while the three surviving pigs from the 15 plasmid group showed a maximum of 10^4^ GEC/mL detectable at Day 7 pc in one sample only (Figure 3A,B). 

### 3.2. Immunization with the 15 Plasmids Induces ASFV-Specific T-Cells That Specifically Recognize M488R

**Figure 4:**
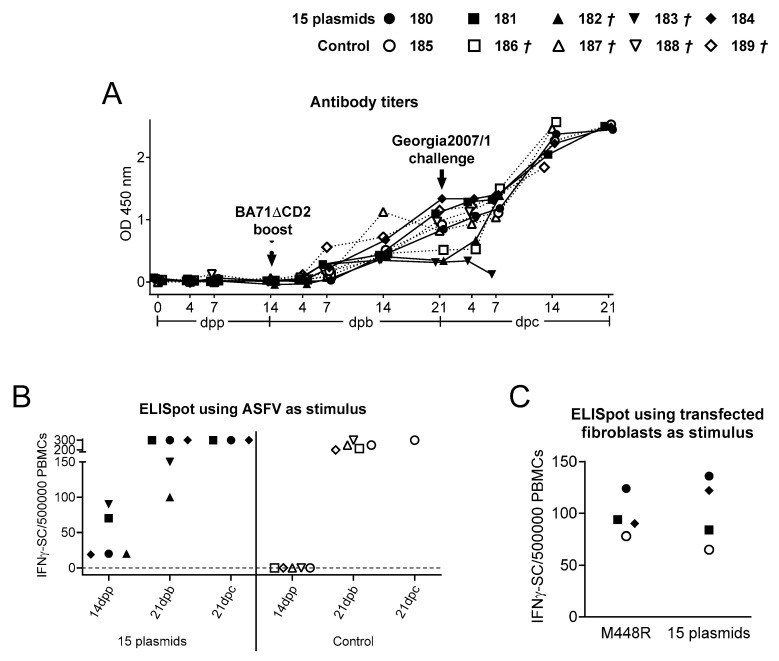
M488R is frequently recognized by ASFV-specific T-cells. (**A**) ASFV-specific antibodies (total IgG) were measured by ELISA, expressing the results as OD values at a wavelength of 450 nm. Solid lines represent the 15-ASFV plasmids group and dashed lines represent the control group. (**B**) ASFV-specific T-cell responses were assessed by IFNΥ ELISpot using PBMCs isolated at different time points: after DNA prime (14 dpp), after BA71∆CD2 boost (21 dpb) and after Georgia2007/1 challenge (21 dpc). (**C**) IFNΥ ELISpot using PBMCs from surviving animals as effector cells and autologous fibroblasts transfected with pCMV-Ub-M448R or the 15 plasmids as specific stimuli. *†* indicates animals succumbing to ASFV challenge.

As expected due to the presence of ubiquitin in the N-terminus of the ASFV proteins [29,34], no antibody responses were detectable after priming with the 15 plasmids (Figure 4A). All pigs seroconverted by Day 14 post-inoculation with BA71ΔCD2, showing no statistically significant differences between both groups at any time post-boosting. In addition, a clear boost of antibody titers after Georgia2007/1 challenge was observed in all animals, reaching their maximum titers at the latest time point investigated (Figure 4A). 

Immunization with the 15 plasmids induced statistically significant ASFV-specific IFNΥ responses, detectable 14 days after the second plasmid injection (Figure 4B). By Day 21 pb (after BA71ΔCD2 boosting), all pigs showed indistinguishable ASFV-specific T-cell responses, from both the 15 plasmids and the control group. Interestingly, the two pigs from the 15 plasmids group that did not survive Georgia2007/1 challenge showed the lowest level of antibodies and ASFV-specific T response at the time of Georgia2007/1 challenge (Figure 4A,B). Surviving pigs did show very strong cellular responses prior euthanasia. 

To identify the specificity of the T-cells recognized by the surviving pigs, PBMCs isolated 21 days after Georgia2007/1 challenge were stimulated in vitro with autologous fibroblasts transfected with each individual recombinant plasmid or a mix containing the 15 plasmids, and the specific IFNΥ secretion was detected by ELISpot (Figure 4C). Interestingly, only the fibroblasts transfected with pCMV-Ub-M448R induced significant IFNΥ responses in all four surviving pigs and the response was similar in magnitude to that obtained with fibroblasts transfected with the mix of the 15 recombinant plasmids (Figure 4C). Interestingly, M448R was also recognized by PBMCs from control pigs, indicating that specific T-cells against this antigen are also induced after BA71∆CD2 inoculation without the need of a DNA priming. 

### 3.3. DNA Immunization with pCMV-Ub-M448R and pCMV-Ub-MGF505-7R Improves Protection against Georgia2007/1 Lethal Challenge

**Figure 5:**
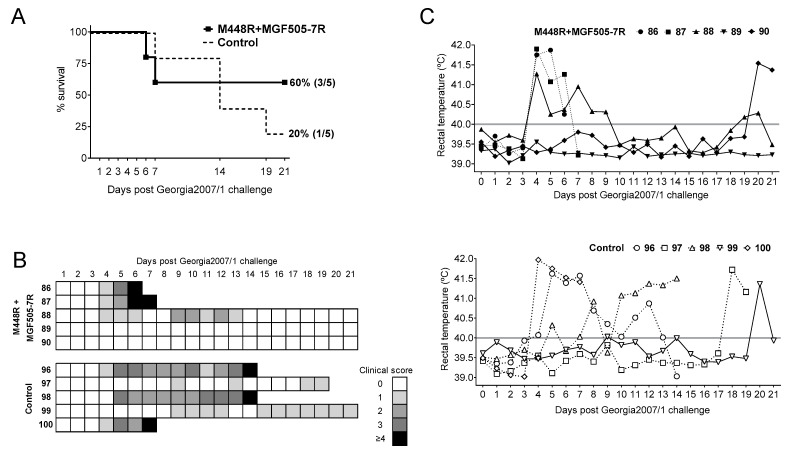
Priming pigs with pCMV-Ub-M448R and pCMV-Ub-MGF505-7R improves the protection afforded by suboptimal BA71ΔCD2 immunization against lethal Georgia2007/1 challenge. Pigs were immunized twice with either the empty pCMV-Ub plasmid (Control) or pCMV-Ub-M488R + pCMV-Ub-MGF505-7R and next boosted with 10^3^ PFU BA71ΔCD2. Two weeks later, all pigs were challenged with a lethal dose of Georgia2007/1 and (**A**) deaths, (**B**) ASF typical clinical signs and (**C**) rectal temperature from pCMV-Ub-M488R + pCMV-Ub-MGF505-7R (top) and control (bottom) groups were recorded daily. Solid lines represent animals that survived the challenge while dashed lines symbolize animals that succumbed to the challenge.

**Figure 6:**
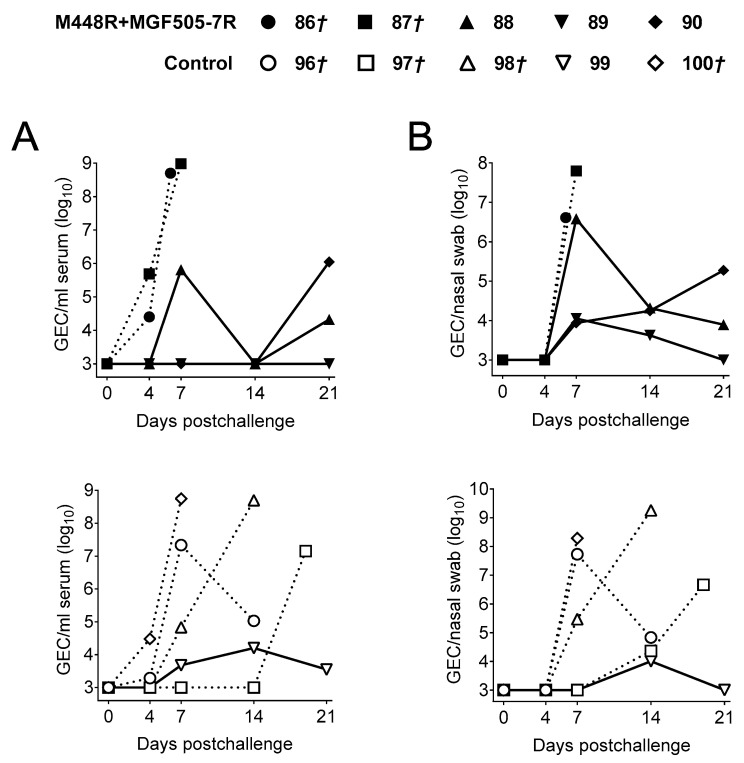
Virus DNA titers found in sera and nasal swabs of pigs after Georgia2007/1 challenge. (**A**) ASFV GEC titers found in sera and (**B**) nasal swabs at different times post Georgia2007/1 challenge detected by qPCR in the pCMV-Ub-M448R and pCMV-Ub-MGF505-7R primed group (top) and the control group (bottom). Solid lines represent animals that survived the challenge while dashed lines symbolize animals that succumbed to the challenge.

Given the promiscuous recognition of M448R by ASFV-specific T-cells, we decided to test the protective potential of a simple DNA formulation based on two plasmids: pCMV-Ub-M488R and pCMV-Ub-MGF505-7R, recently described in vitro as an ASFV CD8^+^ T-cell antigen [23], following the immunization strategy described before (Figure 1). Once again, only one out of the five control pigs (primed twice with pCMV-Ub) survived Georgia2007/1 lethal challenge. On the contrary, three out of the five pigs primed with pCMV-Ub-M488R and pCMV-Ub-MGF505-7R survived (Figure 5A). In this occasion, two out of the three surviving pigs from the immunized group showed no clinical signs compatible with ASF (Figure 5B), with Pig 89 showing no fever at any time point investigated, while Pig 90 showed a peak of fever at the end of the study (Figure 5C). The third surviving pig in the immunized group (Pig 88) recovered from mild apathy and early fever, resolving the infection afterwards. Conversely, the only control pig surviving ASFV lethal challenge (Pig 99) suffered prolonged lethargy starting at Day 9 pc and lasting until the end of the trial. Moreover, it developed cyanosis in ears and tail, even though fever was only detectable at the end of the experiment (Figure 5C). Matching fever and clinical signs, succumbing pigs showed higher virus GEC numbers in both sera (Figure 6A) and nasal swabs (Figure 6B), 3–4 logs above those found in surviving pigs. Only Pig 89 showed no detectable virus in serum at any time after infection.

### 3.4. M448R and MGF505-7R Are Frequently Recognized by ASFV-Specific T-Cells 

**Figure 7:**
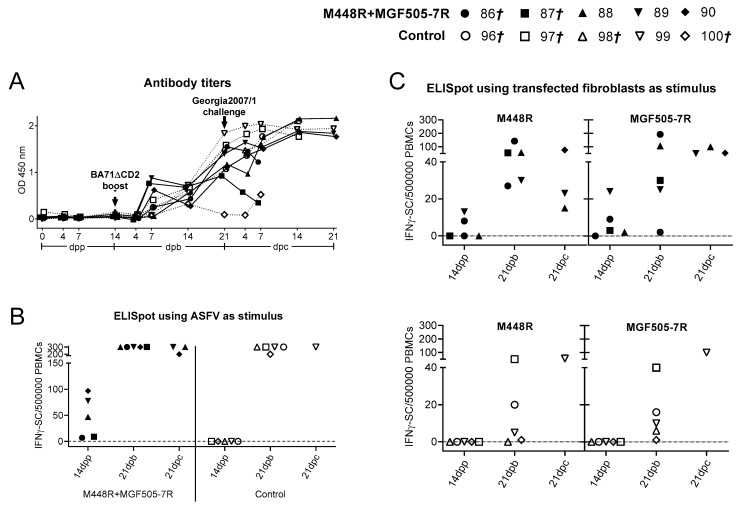
M488R and MGF505-7R are frequently recognized by specific T-cells. (**A**) ASFV-specific antibodies (total IgG) were measured by ELISA, expressing the results as OD values at a wavelength of 450 nm. Solid lines represent the M448R + MGF505-7R group and dashed lines represent the control group. ASFV-specific T-cell responses were assessed by IFNΥ ELISpot using as stimulus (**B**) ASFV or (**C**) autologous fibroblasts transfected with either pCMV-Ub-M448R or pCMV-Ub-MGF505-7R at different time points: after DNA prime (14 dpp), after BA71∆CD2 boost (21 dpb) and after Georgia2007/1 challenge (21 dpc). Data from the M448R+MGF505-7R primed group and control group are represented at the top and bottom, respectively. *†* indicates animals succumbing to ASFV challenge.

Administration of the pCMV-Ub-M448R and pCMV-Ub-MGF505-7R plasmids did not induce any detectable ASFV-specific antibody response in the IgG ELISA (Figure 7A) due to the presence of ubiquitin in their N-terminus [29,34]. Conversely, all pigs but one (Pig 100) showed similar antibody responses by day 21 post BA71∆CD2 boosting. Surviving pigs showed maximum antibody titers after Georgia2007/1 challenge, albeit protection did not perfectly correlate with the levels of antibodies existing at the time of challenge (Figure 7A). 

Priming with pCMV-Ub-M448R and pCMV-Ub-MGF505-7R induced a significant number of T-cells that specifically recognized ASFV in vitro. The number of T-cells detectable after BA71∆CD2 boost dramatically increased in DNA primed pigs, and also in control pigs, reaching similar levels, with the exception of Pig 100 that showed no specific T-cell responses (Figure 7B).

Interestingly, specific IFNΥ responses against M448R and MGF505-7R were detectable in DNA primed pigs before BA71∆CD2 boost, and in both groups of animals after boosting with BA71∆CD2 (Figure 7C, top and bottom). After Georgia2007/1 challenge, surviving animals showed high numbers of T-cells capable to specifically recognize ASFV (Figure 7B), and also the M448R and MGF505-7R ASFV proteins (Figure 7C). 

## 4. Discussion

The socioeconomic impact of ASF ranks this lethal swine disease as the number one challenge for the pig industry worldwide. This relates to animal well-being, trade restriction and consequently availability of food at affordable prices based on pork. As one of the most cost-effective measures, ASF vaccine development has a major priority. Thus far, only LAVs have shown solid protection against ASFV. Even though most of the times protection is limited to homologous protection [13,16,17], LAVs have become essential tools to confirm the protective role of innate [35,36,37,38] and adaptive immune responses, with both humoral response [39,40,41] and ASFV-specific CD8^+^ T-cells [21] playing crucial roles in protection. In this regard, recombinant LAVs are the most advanced candidates against the pandemic virus [14,15,16,17]. Although still pending approval from the authorized governmental agencies, LAVs candidates should contribute in the near future to control ASF in endemic areas and in emergencies. However, it is difficult to envision the use of LAVs in ASF-free areas in the short term, overall taking into account the non-vaccination policy applied against other diseases of obliged declaration to the OIE in disease-free countries. On the other hand, subunit vaccines against ASF are not a utopia, but needing extra research to understand the disease and subsequent development efforts [22,42], including discovery of protective antigens. 

Subunit vaccine formulations used in experimental setups have only conferred partial protection against ASF [29,34,43]. Unfortunately, these results have not been reproduced against the Georgia2007/1 pandemic virus currently circulating [22,44,45]. Confirming this reality, preliminary work perform in our laboratory using DNA immunization as a tool and CD2v as vaccine target failed to confer any protection against Georgia2007/1 challenge (unpublished results), while the same formulation conferred partial protection against E75, a genotype I ASFV strain isolated in Spain in the 1970s [34]. Differences in the amino acid sequence of CD2v between these two strains might partially explain the different extent of protection. Thus, the two CD8^+^ T-cell determinants identified in the E75 strain [34] are absent in the CD2v sequence of the Georgia2007/1 ASFV. Nonetheless, many other reasons might contribute to this outcome, including the differential degree of virulence due to a possible different biology observed for these two ASFV strains. Similar results have also been described for LAV prototypes, showing different protection abilities depending on the ASFV strain used for vaccine designing and for experimental challenge [46,47]. Thus far, only complex formulations encoding multiple ASFV antigens have shown some protective efficacy against experimental lethal Georgia2007/1 challenge, but seldom avoiding the death of the animals [45,48]. Due to the failure of previous formulations using DNA vaccination alone against Geogia2007/1 and aiming to increase the chances to discover new T-cell determinants with protective potential against Georgia2007/1, here we decided to immunize pigs following a prime–boost regime.

Recent efforts performed using a multiple approach that combined in silico predictions, immunopeptidomics and in vitro antigen presentation assays allowed identifying a panel of novel Georgia2007/1 antigens commonly recognized by CD8^+^ T-cells from surviving pigs [23]. Here, we present parallel studies performed in our laboratory confirming immunopeptidomics as a consistent approach not only to identify ASFV CD8^+^ T-cell determinants in vitro, but also to discover novel ASFV antigens with protective potential. Taking into account the failure of our experimental DNA vaccines based on CD2v, here we implemented a heterologous prime–boosting immunization protocol, boosting DNA-immunized pigs with a partially protective dose of 10^3^ PFU of BA71∆CD2 [14] aiming to optimize the protective potential of our DNA vaccine formulations against Georgia2007/1. A similar immunization strategy using vector-expressed ASFV antigens as a prime, followed by a boost with the naturally attenuated OURT88/3 ASFV isolate was previously published [49]. Nevertheless, that specific study was focused on antibody response, and neither cell-mediated response nor protective potential was evaluated. Our heterologous prime–boost protocols were shown to increase the chances to discover ASFV antigens with protective potential for future vaccine formulations in optimized expression vectors. 

In line with previous experiences, priming with DNA vaccines encoding ubiquitinated antigens induced ASFV-specific cellular responses detectable directly after DNA immunization, while no specific antibody response was observed [29,34]. Unexpectedly, no significant differences were observed in the kinetics or in the levels of ASFV-specific IFNΥ-secreting cells after administration of BA71ΔCD2 between DNA-primed and control animals, at least at the time tested (21 dpc) and by using the IFNΥ ELISpot as readout. The consistent improvement in the survival rate of vaccinated animals versus control pigs suggests that the T-cell repertoire induced by the DNA priming contributed to a better control of the infection. It must be noted that the two vaccine formulations successfully tested in the in vivo experiments here described provided the same degree of protection (60% of the pigs survived the ASFV lethal challenge). Although the clinical score of the surviving pigs seemed slightly better in the group primed with pCMV-Ub-M448R + pCMV-Ub-MGF505-7R than with the 15 ASFV clones, the differences have no statistical significance. Moving into the field of speculation, plasmids within the plasmids mix neglected due to the lack of significant responses (data not shown) might have contributed to parallel the protection rates observed when combining pCMV-Ub-M448R and pCMV-Ub-MGF505-7R. The protection capabilities of these two plasmids individually were not tested. 

Combining the parallel approaches described here and in our previous report [23], we aim to identify as many protective determinants as possible in the Georgia2007/1 proteome. Despite the phenotype of the T-cells secreting IFNΥ has not been confirmed, the use of transfected as APCs, in combination with the ubiquitination strategy, points to a CD8^+^ T cell phenotype, as recently described for other antigens using a similar technology [23]. 

The lack of in vitro correlates for ASFV protection complicates providing a definitive explanation of why pigs do or do not survive. In agreement with multiple experiences in our lab and independently of the ASFV strain used for challenge, no perfect correlation existed between protection and the level of antibodies detectable by ELISA or the number of IFNΥ specific T-cells detectable by ELISpot at the time of challenge. On some occasions, this relationship seems to exist, as shown in Figure 4 for the IFNΥ spots found before Georgia2007/1 challenge, but, in many others, pigs with large amounts of specific T-cells and antibodies do not survive ASFV challenge. These results confirm that, together with antigen discovery, extra efforts should be directed at searching for consistent correlates of protection. Solving this gap would not only help rational vaccine design, but also avoid unnecessary suffering to pigs, since up to today no alternative to Georgia2007/1 challenge exists to characterize protective determinants or vaccine prototypes. 

Proteins M448R and MGF505-7R were identified not only as frequently recognized ASFV-specific T-cell determinants, but also as candidates to be included in future and complex vaccine formulations. Even though M448R remains an uncharacterized protein in ASFV [19], orthologs have been described in other dsDNA viruses [50,51]. Their RNA ligase activity is involved in tRNA repair [52], and hence can facilitate host evasion due to bypassing immune response to damaged RNA associated with virus infection [53,54]. M448R sequence is conserved among ASFV isolates [55], a common feature of metabolic enzymes [56]. MGF505-7R ASFV gene (A528R in the old nomenclature), also conserved among ASFV strains [55,57], has been described to encode an IFN inhibitor protein [58]. 

While this is the first report showing the immunogenicity of the combinations of both proteins (MGF505-7R and M448R), M448R was previously included in a complex experimental vaccine formulation based on recombinant viral vectors encoding individual ASFV proteins [59]. In this case, immunization of both NIH dd minipigs and outbred pigs with this cocktail induced specific humoral and T-cell response against M448R. However, the M448R-specific response was not uniform in all pigs and, despite a delayed onset of clinical signs and reduced viremia and viral loads in tissues, all pigs died after lethal challenge with OURT88/1 ASFV (genotype I). Further testing of MGF505-7R and M448R, using the appropriate vaccine vectors would definitively shed more light on their protective potential. These and others results obtained thus far using complex subunit vaccine formulations [43,44,48] confirm the complexity of developing safe and efficient subunit vaccines in the future as well as the need of identifying as many protective antigens as possible within the ASFV. We believe that this is just one piece of a puzzle, which will also need further research to select the optimal combination of expression vectors and immune adjuvants if aiming to mimic the solid protection conferred by LAVs. In addition, a better understanding of protective innate and adaptive immunity is fundamental to optimize in a rational manner vaccine compositions in the future. 

## Figures and Tables

**Table 1:** Selection of ASFV ORFs for in vivo immunization studies.

Peptides	Protein	Activity/Similarity	Plasmid	Anti-FLAG Tag
DSFIPKEYSQSI	B475L	Unknown	pCMV-Ub-B475L-Flag	+
NKKLYEKML				
RKQELLTSQEL				
KVDEFYYKY	B602L	Major capsid protein p72 chaperone	pCMV-Ub-B602L-Flag	+
ITKTFVNNI	p37 (CP2475L/partial)	Structural polyprotein	pCMV-Ub-P37-Flag	+
RSKKDFKQI	D339L	RNA polymerase subunit 7	pCMV-Ub-D339L-Flag	+
YSEKEKETI	DP238L	Unknown	pCMV-Ub-DP238L-Flag	+
NKIKLLNEYL	EP424R	FTS J-like methyl transferase domain	pCMV-Ub-EP424R-Flag	+
NPTIIMEQY	H339R	Unknown	pCMV-Ub-H339R-Flag	+
KNILNTLMF	I226R	Unknown	pCMV-Ub-I226R-Flag	+
NTILTNKI	I243L	Transcription factor SII	pCMV-Ub-I243L-Flag	+
TAKNIKVVI	I73R	Unknown	pCMV-Ub-I73R-Flag	+
YKIYIHSDL	I9R	Unknown	pCMV-Ub-I9R-Flag	+
YIKTSKQEYL	K145R	Unknown	pCMV-Ub-K145R-Flag	+
RAKIPAQEI	M448R	RNA ligase	pCMV-Ub-M448R-Flag	+
YAIHHAPKL	MGF505-1R	Unknown	pCMV-Ub-MGF505-1R-Flag	+
KKYQHKHIL	MGF505-3R	Unknown	pCMV-Ub-MGF505-3R-Flag	+

All SLAI peptides identified by MS-based immunopeptidomics using ASFV-infected PAMs are listed in column Peptides, while the ASFV proteins containing the identified peptides and their putative functions are listed in column Protein and Activity/Similarity, respectively. The plasmids encoded each of the selected ASFV ORFs as fusion with ubiquitin at their N-terminus and with a FLAG-tag in the carboxyl terminal end (listed in the column Plasmid). Immunofluorescence assays using an anti-FLAG antibody allowed detecting the expression of the fusion proteins after transient transfection of each plasmid in RK13 (indicated with a + sign).

## Data Availability

Data is contained within the article.
